# Development of a text mining algorithm for identifying adverse drug reactions in electronic health records

**DOI:** 10.1093/jamiaopen/ooae070

**Published:** 2024-08-16

**Authors:** Britt W M van de Burgt, Arthur T M Wasylewicz, Bjorn Dullemond, Naomi T Jessurun, Rene J E Grouls, R Arthur Bouwman, Erik H M Korsten, Toine C G Egberts

**Affiliations:** Division of Clinical Pharmacy, Catharina Hospital Eindhoven, 5623 EJ Eindhoven, The Netherlands; Division Healthcare Intelligence, Catharina Hospital Eindhoven, 5623 EJ Eindhoven, The Netherlands; Department of Electrical Engineering, Signal Processing Group, Technical University Eindhoven, 5612 AP Eindhoven, The Netherlands; Division Healthcare Intelligence, Catharina Hospital Eindhoven, 5623 EJ Eindhoven, The Netherlands; Department of Mathematics and Computer Science, Technical University Eindhoven, 5612 AP Eindhoven, The Netherlands; Netherlands Pharmacovigilance Centre LAREB, 5237 MH 's-Hertogenbosch, The Netherlands; Division of Clinical Pharmacy, Catharina Hospital Eindhoven, 5623 EJ Eindhoven, The Netherlands; Department of Electrical Engineering, Signal Processing Group, Technical University Eindhoven, 5612 AP Eindhoven, The Netherlands; Department of Anesthesiology, Catharina Hospital Eindhoven, 5623 EJ Eindhoven, The Netherlands; Division Healthcare Intelligence, Catharina Hospital Eindhoven, 5623 EJ Eindhoven, The Netherlands; Department of Electrical Engineering, Signal Processing Group, Technical University Eindhoven, 5612 AP Eindhoven, The Netherlands; Department of Clinical Pharmacy, University Medical Centre Utrecht, 3584 CX Utrecht, The Netherlands; Department of Pharmacoepidemiology and Clinical Pharmacology, Utrecht Institute for Pharmaceutical Sciences, Faculty of Science, Utrecht University, 3584 CX Utrecht, The Netherlands

**Keywords:** adverse drug reaction, text mining, free-text, natural language processing, clinical decision support systems, electronic health record

## Abstract

**Objective:**

Adverse drug reactions (ADRs) are a significant healthcare concern. They are often documented as free text in electronic health records (EHRs), making them challenging to use in clinical decision support systems (CDSS). The study aimed to develop a text mining algorithm to identify ADRs in free text of Dutch EHRs.

**Materials and Methods:**

In Phase I, our previously developed CDSS algorithm was recoded and improved upon with the same relatively large dataset of 35 000 notes (Step A), using R to identify possible ADRs with Medical Dictionary for Regulatory Activities (MedDRA) terms and the related Systematized Nomenclature of Medicine Clinical Terms (SNOMED-CT) (Step B). In Phase II, 6 existing text-mining R-scripts were used to detect and present unique ADRs, and positive predictive value (PPV) and sensitivity were observed.

**Results:**

In Phase IA, the recoded algorithm performed better than the previously developed CDSS algorithm, resulting in a PPV of 13% and a sensitivity of 93%. For The sensitivity for serious ADRs was 95%. The algorithm identified 58 additional possible ADRs. In Phase IB, the algorithm achieved a PPV of 10%, a sensitivity of 86%, and an F-measure of 0.18. In Phase II, four R-scripts enhanced the sensitivity and PPV of the algorithm, resulting in a PPV of 70%, a sensitivity of 73%, an F-measure of 0.71, and a 63% sensitivity for serious ADRs.

**Discussion and Conclusion:**

The recoded Dutch algorithm effectively identifies ADRs from free-text Dutch EHRs using R-scripts and MedDRA/SNOMED-CT. The study details its limitations, highlighting the algorithm's potential and significant improvements.

## Introduction

Adverse drug Reactions (ADRs) are a significant concern in healthcare because they contribute to patient harm and increase healthcare costs.^1^^,^^2^ Recurring ADRs (reADRs), when a medication that was previously stopped due to an ADR is re-prescribed, account for 10%-30% of all ADRs and pose a notable challenge for prevention.^3–6^ To prevent the risk of (re)ADRs, clinical decision support systems (CDSS) have been implemented in electronic health records (EHRs) to alert prescribers.^7^ CDSS need structured ADR data in ADR modules from the EHRs to support these alerts.

However, ADRs are often documented as free text (unstructured data) in clinical notes and are therefore not identifiable using CDSS. Moreover, notes in EHRs are often found to be inaccurate, with a higher error rate compared to paper charts (24.4% vs 4.4%).^8^ Therefore, it is important to bridge the documentation gap between the ADR modules and free text by using text mining (TM) to integrate analyzed unstructured ADR data with CDSS.^9^

Previous studies have demonstrated the ability of TM tools to transform unstructured free text into annotated, meaningful information.^10–13^ Specifically, studies of TM tools for ADR detection have found sensitivities ranging from 58% to 90%.^14–16^ However, these studies focused on specific medications,^17^^,^^18^ ADRs,^19^^,^^20^ notes,^20–28^ or settings,^14^^,^^18^ which limited their scope and enabled identification of only a fraction of ADRs. Four recent literature reviews on this topic have provided strategic overview of the progress that has been made. Challenges highlighted in these studies include lack of publicly available training data,^29^ lack of detailed TM application steps,^30^ no validated assessment tool for clinical TM^30^ or externally validated algorithms^31^ and limited data sharing between healthcare organizations.^32^

Additionally, most studies focused on retrieving ADRs from EHRs based on the English language.^27^^,^^28^ To our knowledge, only 2 studies have retrieved medical information that used Dutch text.^33^^,^^34^ In recent years, TM has seen significant development in handling Dutch text, including the refinement of language models like Dutch-specific BERT models.^35^

In our previous study, we used a general approach to identify ADRs from all free-text available in regular hospital EHRs.^34^ We developed a Dutch algorithm that used manually created rules in a CDSS to identify ADRs from free-text notes in EHRs. However, the CDSS was not specifically developed nor optimized for TM applications. It could only identify the relevant text which contained a possible ADR, not the potential ADR itself.

The aim of this study was to develop a TM algorithm to identify possible ADRs in free-text of Dutch hospital EHRs.

## Methods

### Design and setting

This study used the same EHR patient notes as our previous study. That study, which is described in detail in Wasylewicz et al,^34^ compared a CDSS system’s identification of possible ADRs with a manual review. The data included the complete textual history (ie, EHR notes), excluding scanned or imported documents in the EHR system CS-EZIS (version 5.2, Chipsoft B.V., Amsterdam, The Netherlands), of 45 patients. All patients were hospitalized for at least 24 h in the department of geriatrics (*n *=* *15), internal medicine (*n *=* *15), or oncology (*n *=* *15) of the Catharina Hospital, Eindhoven, The Netherlands, a 696-bed hospital, and all patients were discharged prior to 1 June 2018. The EHR notes often contained multiple entries, such as medical history, physical examinations, additional findings, summaries, and therapeutic plans. The causality of the ADRs was assessed by a clinical pharmacist trained in pharmacovigilance using Naranjo. Only ADRs with a Naranjo score greater than or equal to 1 were included. In total, 326 unique EHR notes containing possible ADRs were detected in the data from relatively large 35 000 notes. Of these, 318 unique EHR notes containing possible ADRs were identified after manual review, of which 63 were potentially serious. The CDSS linked to the EHR system (Gaston Medical, Eindhoven, The Netherlands) identified 187 EHR notes containing possible ADRs (including eight identified only by the CDSS). The CDSS missed 139 of the notes identified by the manual review, and 377 notes were incorrectly identified, resulting in a PPV of 32% and a sensitivity of 57%.

The current study used HIX (version 6.3, Chipsoft B.V., Amsterdam, The Netherlands) for the EHR system. The same data were used and extracted using the same software; however, the previous study extracted the data from CS-EZIS rather than HIX. The study was declared not subject to the Research Involving Human Subjects Act (non-WMO) by the medical ethics committee of the Medical Research Ethics Committees United.

### Development process text mining algorithm

**Figure 1.**
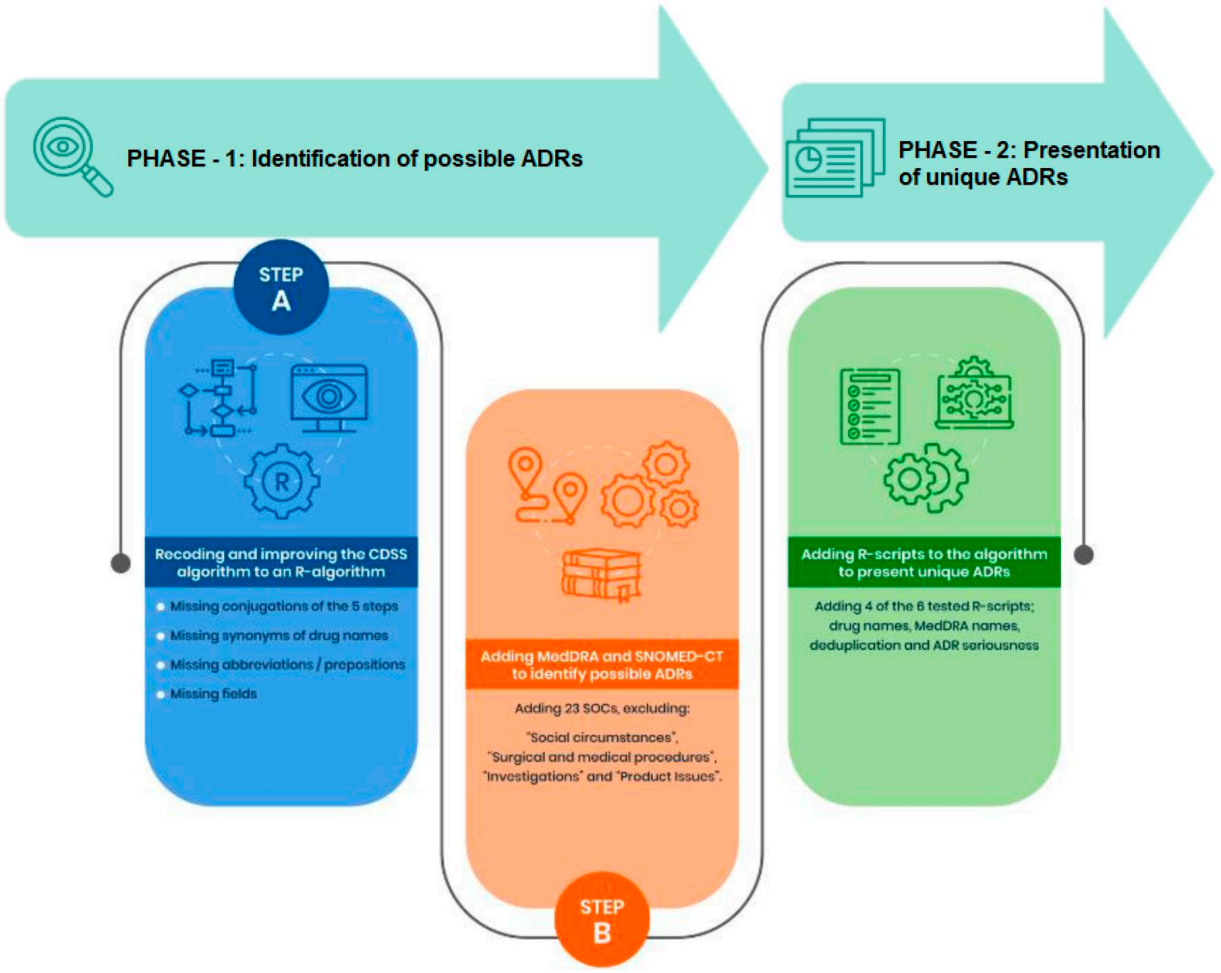
A flowchart of the pipeline for identifying possible ADRs and the phases of this study. The CDSS algorithm was developed in our previous work. In Step A, the CDSS algorithm was recoded, and previously revealed issues were fixed. In Step B, MedDRA and SNOMED-CT medical terms were added to identify possible ADRs. In Phase II, R-scripts were added to present unique alerts.

Figure 1 depicts the two phases and three steps of this study.

#### Phase I: Identification of possible ADRs

##### Step A: Recoding and improving the CDSS algorithm to an R-algorithm

In our previous study, the CDSS identified EHR notes containing possible ADRs using 5 key strategies. To improve future application, in the present study the algorithm developed in the CDSS was reprogrammed to a TM tool (R; The R Foundation, Auckland, New Zealand).^36^ After recoding to R, the algorithm was evaluated whether it had the same performance as our previously used CDSS.

After recoding the CDSS algorithm to R, we improved the algorithm by solving the problems identified in our previous study. The libraries of the 5 strategies were supplemented with positive and negative triggers (ie, keywords implying ADRs; prepositions followed by a drug group, name, brand name, or abbreviation; complication field and registration containing the field “drug-induced”). Following these improvements, the algorithm was run over the 35 000 notes to identify text blocks containing possible ADRs, mimicking the real-world situation.

The text blocks with possible ADRs identified by the algorithm were matched with those from our previous work by one researcher (B.W.M.B). If a match was found, the ADRs were counted as correctly identified. If no match was found, the ADRs found by the algorithm were assessed by two researchers (B.W.M.B. and A.T.M.W.) for technical correctness and clinical relevance. If the ADR was technical correct and clinically relevant, the causality was assessed by a clinical pharmacist trained in pharmacovigilance using Naranjo. Only ADRs with a Naranjo score of ≥1 were included. ADRs found in our previous study and not found by the algorithm were counted as missed ADRs. These data were used as input for PDCA cycles to improve the algorithm sensitivity to >80% for identifying EHR notes containing potentially serious ADRs and to >75% for identifying EHR notes containing any ADRs. The output consisted of text blocks containing possible ADRs.

##### Step B: Adding MedDRA and SNOMED-CT to identify possible ADRs

Possible ADRs were identified in the text blocks generated by step A using the Medical Dictionary for Regulatory Activities (MedDRA version 24.1) terms and the related Systematized Nomenclature of Medicine Clinical Terms (SNOMED-CT), including synonyms. The current MedDRA to SNOMED-CT mapping contains the top 80% of ADRs submitted to the international WHO ADR reporting system. MedDRA contains 27 system organ classes (SOCs), of which 23 were included; “Social circumstances”, “Surgical and medical procedures”, “Investigations” and “Product Issues”, were excluded because they had no relation to ADRs.

The location of the MedDRA lower-level term (LLT), the MedDRA primary term (PT), and the drug name (using the Dutch G-standard database) were identified in the free-text. All punctuation was excluded. Next, the found MedDRA terms and drug names were matched and merged with the outcome of step A. Then, the maximum number of characters (ie, proximity between a MedDRA or SNOMED-CT ADR term and a drug name) was calculated as a PPV for each result, ranging from 1 to 100 (see Supplementary material S1 for the created algorithm). The output consisted of text blocks containing identified possible ADRs with MedDRA and SNOMED-CT terms.

#### Phase II of the pipeline: Adding R-scripts to the algorithm to present unique ADRs

In Phase II, we examined whether existing R-scripts for TM could improve pipeline sensitivity and PPV to at least 50%. Six R-scripts (ie, drug names, MedDRA names, deduplication, DNA/Levensthein, negation, and seriousness of the ADR) were evaluated and tested sequentially. For each R-script, the sensitivity, PPV, and F-measure were calculated. Scripts with an improvement on the F-measure were approved to be utilized.

In the first script, drug names were added and removed to create separate libraries with positive and negative drug names. For the second script, separate libraries were created with positive and negative MedDRA names. Next, the deduplication script was tested to correctly ascertain unique ADRs; all duplicate ADRs (ie, the same ADR occurring on the same drug belonging to the same patient) were removed. Then, the DNA script (also known as Levensthein) was evaluated. This script contained all MedDRA and drug names and modified or added these names with one letter. For example, a common typo for the gold standard “trimetoprim” is “trimet***h***oprim”. Next, the negation script was tested. This script contained four Dutch negation words: “geen” (“none”), “niet” (“not”), “niks” (“nothing”), and “nooit” (“never”). These words had to be within a distance of 10 characters from the MedDRA term to be ejected. The last script used the MedDRA Important Medical Event list to classify the seriousness of the ADRs. The output of this phase consisted of unique identified ADRs with concrete drug event association.

### Data collection

The data collected for each ADR included the symptoms/reaction, involved medication, trigger word, MedDRA term, surrounding paragraph/context, date, form name and registering healthcare professional. If an ADR was registered in the EHR module, the status of the ADR being approved by a physician or a pharmacist and the severity of the ADR were also documented. The anonymized data were recorded, edited and saved using Research Manager^®^ (Cloud9, Deventer).

The sensitivity, PPV, and F-measure of the algorithm were calculated. Sensitivity (ie, the ability to identify true positive cases) was equal to the number of true positives divided by the sum of true positives and false negatives. PPV (ie, the likelihood that the algorithm corresponds to a true-positive case) was equal to the number of true positives divided by the total number of cases identified by the algorithm (true positives and false positives). F-measure, a measure of a test's accuracy, was calculated from the PPV and sensitivity of the test. The highest possible F-measure value was 1.0, indicating perfect sensitivity and PPV, and the lowest possible value was 0, indicating that either the sensitivity or the PPV is zero.

## Results

### Patient and data characteristics

Patient and data characteristics are described in Wasylewicz et al.^34^ The mean age of the patients was 68 years (range 21-92), and 64.4% were female. The Charlson Comorbidity Index score was 5 (range 0-13). A median of eight (range 0-20) different medications were used. Patients had a median of 3 hospitalizations (range 1-39) and 60 ambulant visits (ie, hospital stay <24 h) (range 2-433), resulting in a median medical history of 7.4 years (range 0.01-18). The median number of free-text EHR notes per patient was 585 (range 41-2820) with approximately 35 000 free-text EHR notes for review and surpassing 3 million words.

#### Phase I: Identification of possible ADRs

##### Step A: Recoding and improving the CDSS algorithm to an R-algorithm

In our previous study, a total of 326 unique EHR notes containing possible ADRs from 35,000 notes were identified, 318 by manual review and an additional eight exclusively identified by the CDSS.

After recoding the algorithm to R, the algorithm performed similar to the CDSS. The algorithm identified 97% (*n* = 182) of the EHR notes containing possible ADRs found by the CDSS. Five EHR notes containing possible ADRs were missed; three were missed due to typos in the EHR database and two because of an EHR conversion from CS-EZIS to HIX.

Next, we worked to improve the algorithm based on previously identified search strategy issues in Wasylewicz et al.^34^ After a number of PDCA cycles, the algorithm identified 92% (*n *=* *299) of the EHR notes containing possible ADRs. Every PDCA cycle contributed to achieving a higher sensitivity and a lower PPV. Another 27 were missed, and 2003 were incorrectly identified by the algorithm. The last PDCA cycle achieved a sensitivity of 92% (95% for potentially serious ADRs), a PPV of 13%, and an F-measure of 0.23. We reached the stated targets of sensitivity >80% in identifying EHR notes containing potentially serious ADRs and >75% in identifying EHR notes containing any ADRs.

**Figure 2.**
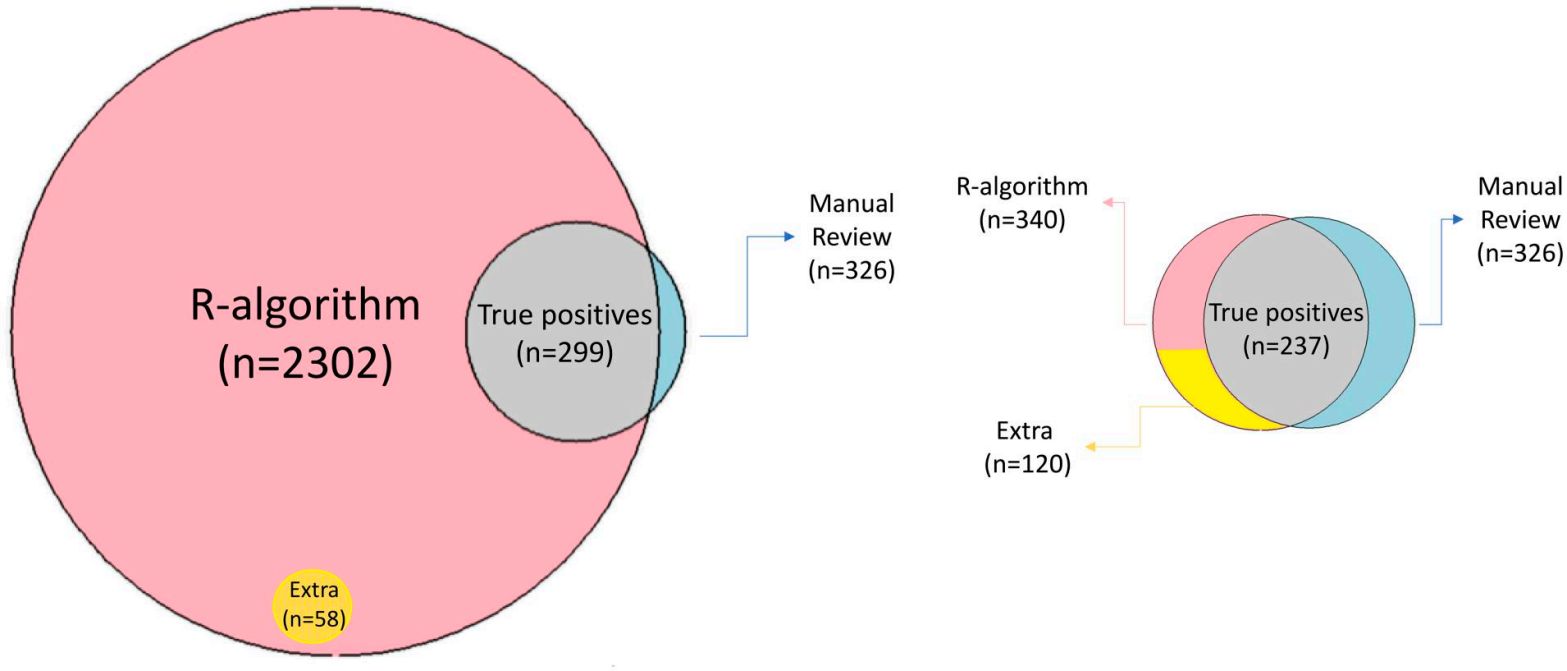
Figure (A) shows the Venn diagram after Phase I step A. Figure (B) shows the Venn diagram after Phase II. The pink circle represents the unique EHR notes containing possible ADRs identified by the R-algorithm. The blue circle represents the unique EHR notes containing possible ADRs found in the manual review. The overlap represents the number of true positives. The yellow circle represents unique EHR notes containing possible ADRs that were found by the R-algorithm and were missed in the manual review.

The algorithm identified 64 additional possible ADRs that were not identified in Wasylewicz et al.^34^ Of these, 58 had a Naranjo score greater than 1 (see Figure 2). The median Naranjo score for the included ADRs was 4 (range 1-6), 24 of them scored probable (score 5-8) and no ADRs were scored definite (score ≥ 9). Results of every step of the pipeline are shown in Table 1.

**Table 1.** The PPV (in %), sensitivity (in %) and F-measure of every step of the pipeline.

	Phase I		
	Step A	Step B	Phase II
**PPV (in %)**	13	10	70
**Sensitivity (in %)**	93	86	73
**F-measure**	0.23	0.18	0.71

##### Step B: Adding MedDRA and SNOMED-CT to identify possible ADRs

The maximum number of characters between a MedDRA or SNOMED-CT ADR term and a drug name was calculated at 43. Some ADRs could not be identified with the algorithm using MedDRA, SNOMED-CT, and synonyms because they did not have an exact LLT code match. The algorithm correctly identified 280 notes containing possible ADRs, 46 were missed, and 2495 were incorrectly identified by the algorithm. This resulted in a sensitivity of 86%, a PPV of 10%, and an F-measure of 0.18.

#### Phase II of the pipeline: Adding R-scripts to the algorithm to present unique ADRS

In Phase II, we evaluated 6 R-scripts to identify and present unique ADRs. Four R-scripts—drug names, MedDRA/SNOMED-CT, deduplication, and serious ADR—improved the overall sensitivity and/or PPV of the pipeline. The biggest improvement in sensitivity (to 97%) was achieved after implementing the drug names and MedDRA/SNOMED-CT scripts. The highest improvements in F-measure and PPV were achieved by the deduplication script, which removed all duplicate ADRs. A combination of the drug names, MedDRA/SNOMED-CT, and deduplication R-scripts resulted in a PPV of 70%, a sensitivity of 73%, and an F-measure of 0.71 (see Table 1 and Figure 2). We achieved a sensitivity of 63% for potentially serious ADRs. The stated PPV target of at least 50% was reached. Supplementary material S2 shows the PPV, sensitivity, and F-measure of the identified possible ADRs at every stage in the pipeline.

### Algorithm outcome

**Figure 3.**
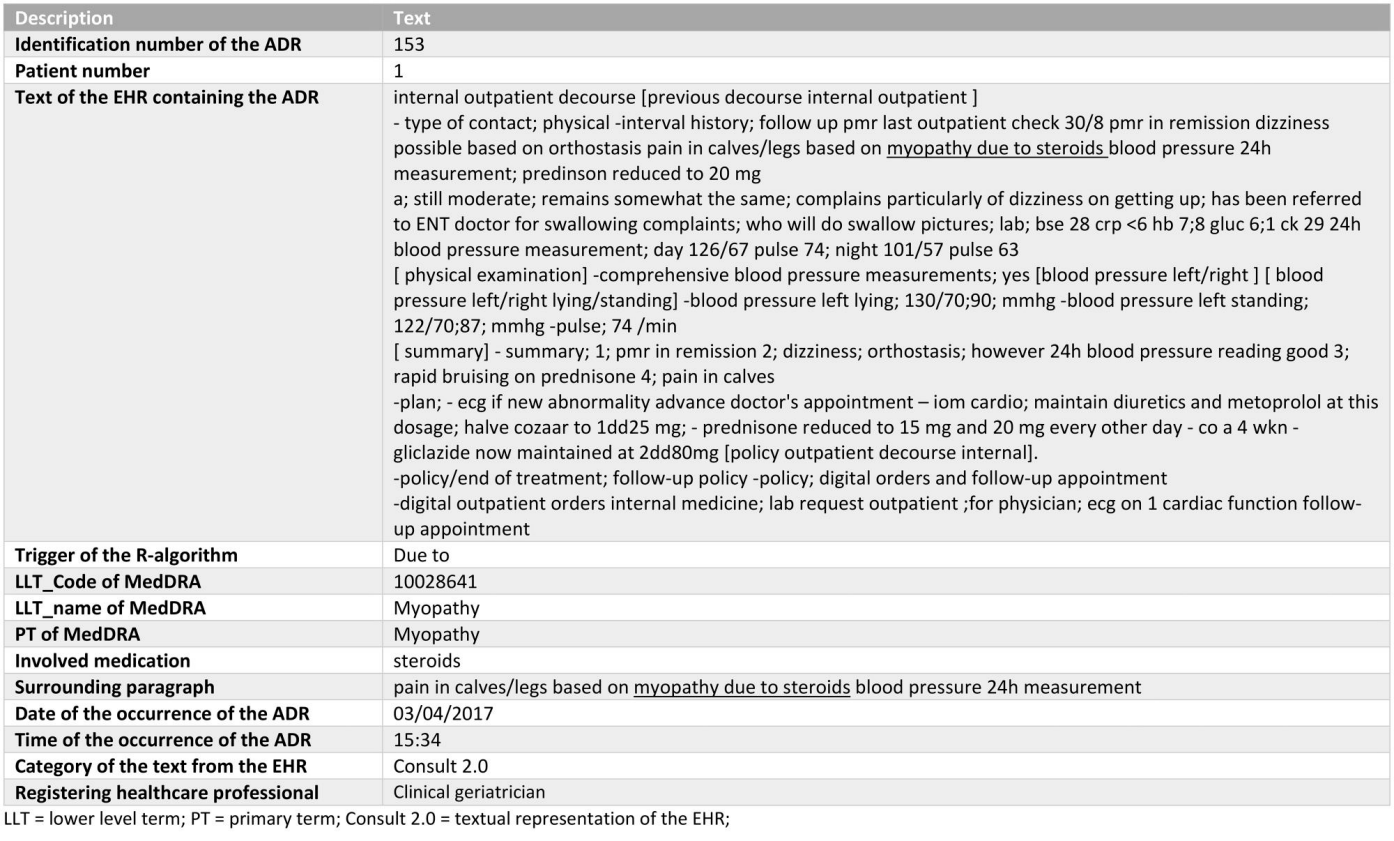
Output of the final R-algorithm (translated from Dutch to English).

Figure 3 shows the output of the algorithm, a notification consisting of a table containing the patient number, trigger of the algorithm, text of the EHR containing the ADR, surrounding paragraph, category of text from the EHR (eg, Consult 2.0, microbiology report), involved medication, LLT term and code of MedDRA, registering healthcare professional and the date and time of the occurrence of the ADR.

## Discussion

The aim of this study was to develop an algorithm in R to identifying possible ADRs using free-text from Dutch hospital EHRs that has the potential to be combined with a CDS system. This study includes strategies for identifying all types of ADRs and ADR symptoms in all types of free-text EHR notes. This study uses SNOMED-CT and MedDRA terminology to identify the ADR symptoms and uses Naranjo to assess the causality of the ADRs.

Compared to our previous study, Wasylewicz et al,^34^ the algorithm improves the PPV from 32% to 70% and the sensitivity from 57% to 73%. We attribute these improvements to the fact that the algorithm developed this study identified possible ADRs using SNOMED-CT and MedDRA terms and included R-scripts to search for symptoms associated with ADRs. An additional 58 possible ADRs were identified in Phase I, compared to the previous study. The algorithm's strong ability to identify possible ADRs can be attributed to its ability to read and analyze free-text data, whereas our previously used CDSS was a rule-based system designed to process structured data.

The current study results indicate that in Phase I, after recoding and improving the algorithm and adding MedDRA/SNOMED-CT terms to the R-script, a PPV of 10% and sensitivity of 86% were reached. To increase these measures in Phase II, several R-scripts were tested, and four scripts were included and two excluded from the algorithm. The deduplication script had the highest improvement on F-measure and PPV, likely because of the “cut-and-paste” feature in the Dutch EHR. The overall sensitivity was 73%, and the overall PPV of the ADR trigger tool was 70%, indicating that more than half of the ADRs were found by the algorithm; therefore, nearly three-quarters of the triggers were considered ADRs.

In Phase II, more possible ADRs were found by the algorithm that were missed in the manual review, likely due to the usage of PT and LLT from MedDRA. For example, an ADR for the same patient regarding “myopathy due to steroids” was written in the EHR in multiple ways: (1) “pain in the muscle” (PT “pain” associated with the SOC “General disorders and administration site conditions”); (2) “myopathy” (associated with the SOC “Musculoskeletal and connective tissue disorders”). No exact match with the manual review was found; however, the algorithm identified the ADR in another way. See Supplementary material S3 for more examples.

The use of ADR trigger tools has proven a valuable and effective strategy to improve ADR detection. The Institute for Healthcare Improvement and the Institute of Medicine have recommended these tools for the detection of hospital-acquired adverse events.^37^^,^^38^ A high PPV is critical for ADR trigger tools to achieve a positive balance between reviewing signals and identifying actual ADRs. There is no universally accepted definition of “good” or “poor” trigger tool performance; however, a PPV of at least 20% is generally considered good.^39^ In this study, the algorithm performed better than the stated PPV target of at least 50% (ie, at least half of triggers are true), which indicates that it is usable in clinical practice. The algorithm could be a useful asset for healthcare professionals because they could use it to discuss ADRs with patients and register them in the appropriate modules.

Comparing the algorithm to other algorithms developed in other studies, their performances depend on various factors, such as dataset, EHR systems, language and the used algorithms. Iqbal and colleagues used a rule-based ADR detection and classification pipeline to extract 19 ADRs specific to antipsychotics and antidepressants from free-text EHRs, with an F-measure of 0.83.^18^ The F-measure for the algorithm in our study was lower at 0.71. This discrepancy may be because the algorithm in this study did not focus on specific ADRs or drug names but used all ADR synonyms and all known drug names, therefore increasing the risk of false positives.

The algorithm's performance was comparable to the higher end of the PPV of the algorithm developed by Hazlehurst et al, who customized MediClass to detect possible adverse events related to vaccines using electronic medical records achieving a PPV ranging from 31% to 74%.^17^ In fact, our algorithm did not lose PPV due to using such a wide approach. Both studies point to the potential utility of algorithms such as the algorithm in identifying ADRs using free-text data.

Another study by Combi and colleagues describes the Magicoder, which also used MedDRA terms to detect ADRs, and achieved a sensitivity of 86.9% and a PPV of 91.8%.^28^ The reason for the lower performance of our algorithm is likely due to differences in methodology between the studies. Magicoder's approach involved initially identification of possible ADRs using an algorithm, followed by an assessment based on a golden standard for accuracy. However, this approach did not include the complete free-text notes. As a result, ADRs that were not detected by the keyword search tool, would not be assessed using the golden standard.

Despite the promising results, the algorithm's performance should be interpreted with caution as the results depend on the dataset and the modelling methods used. A systematic review by Yasrebi-de Kom and colleagues has demonstrated that different algorithms for identifying ADRs using EHRs vary in performance. The review identified 25 studies that used different methods to identify ADRs from EHRs, with an area under the precision recall curve (AUC-PR) of 0.44, concordance of 0.68, area under the receiver operating characteristic curve (AUC-ROC) ranging from 0.71 to 0.99, and accuracy ranging from 0.74 to 0.86.^40^

### Limitations

Although the algorithm showed promising results, it also has limitations. A false positive analysis indicated several improvements based on decisions made in this study. For example, a previous recommendation was to include the term “dd” (“times per day” in Dutch; for example, “2 dd 1 pill” means “one pill two times per day”) as a keyword to imply an ADR. However, use of this keyword resulted in a PPV less than 0.1%, so it was not included in the final algorithm. Additionally, not all MedDRA SOCs were included because some are unrelated to ADRs. Another recommendation was to include all MedDRA and SNOMED-CT terms within 16 characters of the drug name. However, a statistical test indicated that the preferred distance was 43. The false positive analysis showed that more TM techniques are needed to understand the context of trigger words, such as recognizing when effects are considered positive. The R-script used to identify negative trigger words was excluded from this study because it lowered the PPV. The inclusion of positive trigger words could be useful to understand the context of ADRs.

After analyzing false negatives, there were further opportunities to refine the algorithm, such as expanding the library with synonyms for ADRs and drug names and re-evaluating the distance between the drug name, trigger word, and ADR term. The evaluation in this study was conducted with 326 ADRs, and it is possible that more ADRs could be missed when using this algorithm in a sample with a greater number and variety of ADRs.

An important limitation of our study is the exclusion of scanned or imported documents. The EHR system was implemented in stages, launching in 2008 and adopting paperless recording from 2015 onwards. Medical records before 2008 and correspondence between first and second line are available (as scanned PDFs) as part of the multimedia module. Consequently, a significant part of patients' past medical history contained on these forms was not included in our analysis. This limitation may affect the completeness and accuracy of patient and ADR data in our study.

Another limitation of the study was the identification strategies used, which were based on EHRs originating from 45 patients in a single hospital using one EHR system. These 45 patients were in three different internal medicine wards; in our experience, more information is written in the EHR in internal medicine wards than surgical wards, and therefore the dataset may have contained a greater than usual amount of free text. These limitations may have resulted in a failure to discover key identification strategies and overestimation of the tool’s performance. Nevertheless, the EHR history contained notes from ambulant visits and hospitalizations related to several medical specialties (*n* = 12), and the ADRs were recorded by numerous diverse healthcare professionals *(n* = 206).

Naranjo was used to assess causality of the ADRs. However, there is debate on the reliability of this and other algorithms to assess causality due to problems with reproducibility and validity, and “no method is universally accepted for causality assessment of ADRs”.^41^ Moreover, only 24 ADRs had a probable score and none were scored definite. However, Naranjo was still the preferred method in this study for causality assessment.

### Challenges

The main challenges in this field are to establish: (1) a generally accepted definition of tool performance, (2) a robust algorithm for detecting ADRs, (3) an open source database, and (4) a standard for exchanging information. In summary, collaboration and standardization are necessary to create the most effective ADR detection algorithm.^42^

In recent years, Natural Language Processing (NLP) has significantly advanced in extracting relevant information from free text in medical records for different purposes including identifying ADRs from clinical notes. Advances in machine learning, particularly deep learning algorithms have enhanced the ability to process and understand unstructured data. Techniques like Named Entity Recognition (NER) and sentiment analysis have been instrumental in extracting ADR-related information from EHRs.^29^^,^^31^ Pre-trained models like BERT (Bidirectional Encoder Representations from Transformers) have further improved accuracy by understanding context more effectively. Our algorithm incorporated NLP techniques for data preparation, it utilized regular expressions and identified specific information blocks such as drug names and SNOMED/MedDRA terms through NER. Some new studies have employed BERT, but these achieved a comparable performance with F-scores between 0.65 and 0.73.^43^^,^^44^ Only 1 study of Hussain et al had a higher F-score compared to this study, but that only tested non-EHR notes like Twitter and Pubmed.^45^ However, most of these algorithms have not yet been externally validated, prospectively tested or put into production in a hospital. This is because these models need too much processing time to implement as a real-time task in clinical practice.^31^

To study the portability of the algorithm, internal and external validation must be performed. Internal validation takes place in the same hospital with different patients in different wards, whereas external validation takes place in another hospital with different patients, EHRs, healthcare professionals, and/or languages. Assessing the usability of the algorithm in a hospital setting requires investigation into the clinical relevance of the possible ADRs. This is crucial to minimize the number of alerts issued to healthcare professionals, as many alerts are overridden.^34^^,^^46^ While this paper categorizes ADRs into all ADRs and potentially serious ones, it's important to acknowledge that clinical relevance is not solely determined by the severity of the ADR. Additionally, further along the treatment pathway another alert can be issued if the physician initially took no action. For example, if a patient is admitted with hyperkalemia and no action is taken, an alert may still be triggered later when starting a potassium-boosting drug or if potassium levels remain high.

The final step to enhance this algorithm involves integrating it with a system that can give the information back to the healthcare professional, to form an advanced AI system. For example, the EHR or a CDSS. CDSS retrieves structured ADR data, such as the specific drugs used during allergic reactions. This study focused on class names like “antibiotic” rather than specific drugs like “penicillin” or “amoxicillin.” A review by van de Burgt and colleagues shows that combining a CDSS and an algorithm effectively delivers the right information to the right person at the right time.^9^ However, incorporating a “human in the loop” ensures professional acceptance. In summary, the algorithm will integrate with the CDSS, which, upon a patient’s hospitalization, will trigger the algorithm to analyze patient notes and identify potential ADRs. These identified ADRs will then be refined by the CDSS, specifying general terms to specific drugs. A healthcare professional will review and verify the potential ADR with the patient, and an entry will be recorded in the patient’s ADR table within the EHR. This integration helps prevent (re)ADRs by combining unstructured and structured EHR data. An additional challenge is first-line communication (ie, between various EHRs).

## Conclusion

In conclusion, this algorithm is a freely available and effective tool for identifying possible ADRs from free-text Dutch EHRs using MedDRA/SNOMED-CT terms. The algorithm showed a significantly better match with manual review than the currently used CDSS application, and it can be optimized prior to integration with CDSS in a clinical application. Further studies are needed for internal and external validation of the performance of the algorithm.

## Supplementary Material

ooae070_Supplementary_Data

## Data Availability

The data underlying this article are available in the article and in its online supplementary material.
