# Supplementary material for: Development of a text mining algorithm for identifying adverse drug reactions in electronic health records
Source: JAMIA Open. 2024 Aug 16;7(3):ooae070. doi: 10.1093/jamiaopen/ooae070 (PMC11328534; doi:10.1093/jamiaopen/ooae070)
Supplement: ooae070_Supplementary_Data [file ooae070_supplementary_data.zip › SUPPLEMENT 3.docx]

**SUPPLEMENT 3: EXAMPLES OF THE ALGORITHM WELL DETECTED AND NOT WELL DETECTED ADVERSE DRUG REACTIONS**

Example 1:

An ADR for the same patient regarding “abdominal pain due to ibuprofen” was written in the EHR in multiple ways: 1) “pain in the belly” (PT “pain” associated with the SOC “General disorders and administration site conditions”); 2) “abdominal pain” (associated with the SOC “Gastrointestinal disorders”). No exact match with the manual review was found; however, the algorithm identified the ADR in another way.

Example 2:

An ADR for the same patient regarding “to low blood sugar due to insulin” was written in the EHR in multiple ways: 1) “low blood sugar” (associated with the SOC “Investigations”); 2) “hypoglycemia” (associated with the SOC “Endocrine disorders” or “Metabolism and nutrition disorders”). No exact match with the manual review was found; however, the algorithm identified the ADR in another way.
