# Supplementary material for: Development of a text mining algorithm for identifying adverse drug reactions in electronic health records
Source: JAMIA Open. 2024 Aug 16;7(3):ooae070. doi: 10.1093/jamiaopen/ooae070 (PMC11328534; doi:10.1093/jamiaopen/ooae070)
Supplement: ooae070_Supplementary_Data [file ooae070_supplementary_data.zip › SUPPLEMENT 2.docx]

**SUPPLEMENT 2: THE PPV, SENSITIVITY AND F-MEASURE OF THE IDENTIFIED POSSIBLE ADRS OF EVERY STAGE IN THE PIPELINE**

| **Step nr.** | **Step description** | **Improving** | **PPV** | **Sensitivity** | **F-measure** | **Included** |
| --- | --- | --- | --- | --- | --- | --- |
| 1 | Drugnames (deletion and adding) | Sensitivity | 11% | 94% | 0.20 | Yes |
| 2 | MedDRA/ SNOMED-CT terms (deletion and adding) | Sensitivity | 1.6% | 97% | 0.33 | Yes |
| 3 | Deduplication | PPV | 70% | 73% | 0.71 | Yes |
| 4 | DNA script / Levensthein distance | Sensitivity | 1.3% | 95% | 0.02 | No |
| 5 | Negation | PPV | 67% | 60% | 0.62 | No |
| 6 | Serious ADR | PPV | 71% | 63% | 0.69 | Yes |
