# Supplementary material for: Development of a text mining algorithm for identifying adverse drug reactions in electronic health records
Source: JAMIA Open. 2024 Aug 16;7(3):ooae070. doi: 10.1093/jamiaopen/ooae070 (PMC11328534; doi:10.1093/jamiaopen/ooae070)
Supplement: ooae070_Supplementary_Data [file ooae070_supplementary_data.zip › Graphical abstract High resolution.pptx]

## Slide 1
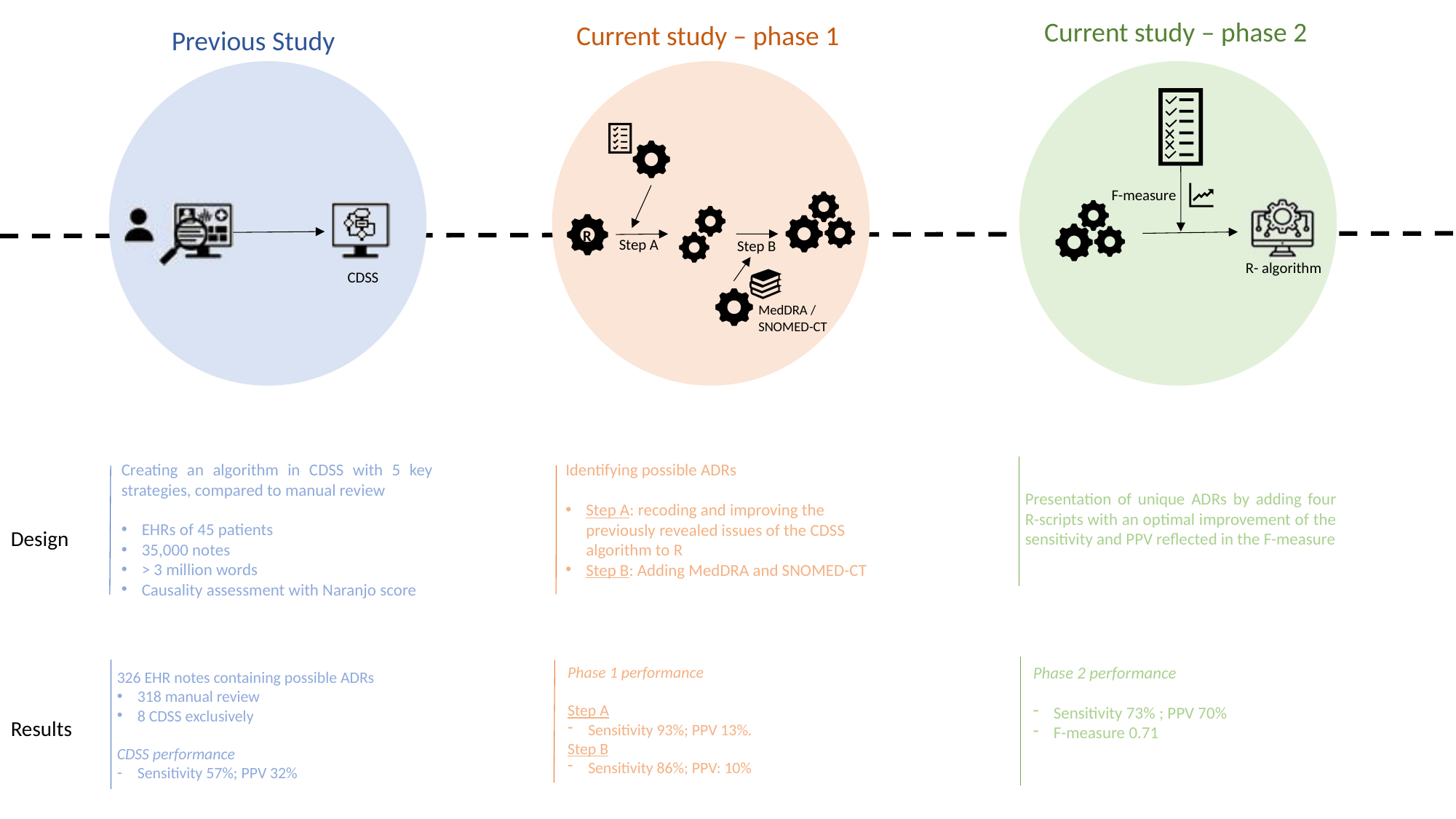

Current study – phase 2
Current study – phase 1
Previous Study
F-measure
R
Step A
Step B
R- algorithm
CDSS
MedDRA /
SNOMED-CT
Creating an algorithm in CDSS with 5 key strategies, compared to manual review
EHRs of 45 patients
35,000 notes
> 3 million words
Causality assessment with Naranjo score
Identifying possible ADRs
Step A: recoding and improving the previously revealed issues of the CDSS algorithm to R
Step B: Adding MedDRA and SNOMED-CT
Presentation of unique ADRs by adding four R-scripts with an optimal improvement of the sensitivity and PPV reflected in the F-measure
Design
Phase 1 performance
Step A
Sensitivity 93%; PPV 13%.
Step B
Sensitivity 86%; PPV: 10%
Phase 2 performance
Sensitivity 73% ; PPV 70%
F-measure 0.71
326 EHR notes containing possible ADRs
318 manual review
8 CDSS exclusively
CDSS performance
Sensitivity 57%; PPV 32%
Results
