# Supplementary material for: Development of a text mining algorithm for identifying adverse drug reactions in electronic health records
Source: JAMIA Open. 2024 Aug 16;7(3):ooae070. doi: 10.1093/jamiaopen/ooae070 (PMC11328534; doi:10.1093/jamiaopen/ooae070)
Supplement: ooae070_Supplementary_Data [file ooae070_supplementary_data.zip › SUPPLEMENT 1.docx]

**SUPPLEMENT 1: THE R-ALGORITHM**

**Fase I step A:**

library(pillar)

library(pacman)

library(tidyr)

library(dplyr)

library(ggplot2)

library(ggfortify)

library(caret)

library(lubridate)

library(readxl)

library(tm)

library(stringr)

library(plyr)

library(tibble)

library(stringdist)

library(stringr)

library(RColorBrewer)

library(zoo)

library(readxl)

### LOAD ALL DATA

options(stringsAsFactors = FALSE)

Database <- read_excel("Database.xlsx")

DatabaseFP <- read_excel("Database.xlsx",

                         sheet = "False positives")

DatabaseO <- read_excel("Database.xlsx",

                        sheet = "CDSS")

### PREP REVIEWER DATA #######################################################################################

colNamesR <- names(Database)

colNames <- colNamesR

colNames <- colNames[colNames != "Verwijderd"]

df <- Database[colNames]

df['PatientNr'] <- na.locf(df['PatientNr'])

df['ADRid'] <- na.locf(df['ADRid'])

df$ID <- cumsum(!duplicated(df[1:2]))

df$`Tgv andere ADR` <- df$ID

agg <- aggregate(ADRAr_ADRAr_omring_paragraaf~ID, data = df, paste0, collapse=" ")

df <- merge(df, agg, by = "ID", all = T)

df$ADRAr_ADRAr_omring_paragraaf <- NULL

df$ADRAr_ADRAr_omring_paragraaf.x <- NULL

df$mergedText <- df$ADRAr_ADRAr_omring_paragraaf.y

agg <- aggregate(ADRAn_ADRAn_omring_paragraaf_1~ID, data = df, paste0, collapse=" ")

df <- merge(df, agg, by = "ID", all = T)

df$label <- "TP_REVIEW"

df$ADRAn_ADRAn_omring_paragraaf_1 <- NULL

df$ADRAn_ADRAn_omring_paragraaf_1.x <- NULL

df$mergedText_1 <- df$ADRAn_ADRAn_omring_paragraaf_1.y

df$mergedText <- ifelse(is.na(df$mergedText), df$mergedText_1, df$mergedText)

df$mergedText_1 <- NULL

df <- df[!is.na(df$`ADR?`),]

df[df$CDSS != 0,]$label <- "TP_GASTON"

df_review <- df

### PREP DATA GASTON #######################################################################################

colNamesFP <- names(DatabaseFP)

colNames <- colNamesFP[colNamesFP != "Verwijderd"]

df <- DatabaseFP[colNames]

df['PatientNr'] <- na.locf(df['PatientNr'])

df['ADRid'] <- na.locf(df['ADRid'])

df$ID <- cumsum(!duplicated(df[1:2]))

df$`Tgv andere ADR` <- df$ID

agg <- aggregate(ADRST_ADRST_paragraaf~ID, data = df, paste0, collapse=" ")

df <- merge(df, agg, by = "ID", all = T)

df$ADRST_ADRST_paragraaf <- NULL

df$ADRST_ADRST_paragraaf.x <- NULL

df$mergedText <- df$ADRST_ADRST_paragraaf.y

df$label <- "FP_gaston"

df <- df[!is.na(df$`ADR?`),]

df_FP <- df

### PREP DATA CDSS ONLY #######################################################################################

colNamesO <- names(DatabaseO)

colNames <- colNamesO[colNamesFP != "Verwijderd"]

df <- DatabaseO[colNames]

df['PatientNr'] <- na.locf(df['PatientNr'])

df['ADRid'] <- na.locf(df['ADRid'])

df$ID <- cumsum(!duplicated(df[1:2]))

df$`Tgv andere ADR` <- df$ID

agg <- aggregate(ADRST_ADRST_paragraaf~ID, data = df, paste0, collapse=" ")

df <- merge(df, agg, by = "ID", all = T)

df$ADRST_ADRST_paragraaf <- NULL

df$ADRST_ADRST_paragraaf.x <- NULL

df$mergedText <- df$ADRST_ADRST_paragraaf.y

df$label <- "ONLY_GASTON"

df <- df[!is.na(df$`ADR?`),]

df_O <- df

### COMBINE DATAFRAMES ##############################################################################

colNamesR <- names(df_review)

colNamesFP <- names(df_FP)

colNamesO <- names(df_O)

colNamesCombined <- intersect(colNamesR, colNamesFP)

colNamesCombined <- intersect(intersect(colNamesR, colNamesFP),colNamesO)

df_review_prune <- as.data.frame(df_review[colNamesCombined], stringsAsFactors = FALSE)

df_FP_prune <- as.data.frame(df_FP[colNamesCombined], stringsAsFactors = FALSE)

df_O_prune <- as.data.frame(df_O[colNamesCombined], stringsAsFactors = FALSE)

df_review_prune <- data.frame(sapply(df_review_prune, as.character), stringsAsFactors = FALSE)

df_FP_prune <- data.frame(sapply(df_FP_prune, as.character), stringsAsFactors = FALSE)

df_O_prune <- data.frame(sapply(df_O_prune, as.character), stringsAsFactors = FALSE)

### REMOVE O FROM FP

df_FPO_prune <- merge(df_FP_prune, df_O_prune, by=c('PatientNr','ADRid'))

df_combined <- rbind(df_review_prune, df_FP_prune,df_O_prune)

### FIND Allergies based on keywords #################################################################################################

### LOAD ALL NIETZEGGENDE WOORDEN

remove(exclusie)

for(f in list.files("../data/bestandenexclusie", pattern=".xlsx", full.names=TRUE)) {

  if (exists("exclusie")){

    exclusie <<-rbind(exclusie,read_xlsx(

      path = f, na = "NULL", skip = 0, col_names = 'words'))

  } else {

    exclusie <<-read_xlsx(

      path = f, na = "NULL", skip = 0, col_names = 'words')

  }

}

for (e in 1:nrow(exclusie)){

  exclusie$words[e] <-gsub("\\", ";",exclusie$words[e], fixed=TRUE)

  exclusie$words[e] <-str_replace_all(exclusie$words[e], "[?]", ";")

  exclusie$words[e] <- tolower(exclusie$words[e])

}

exclusie <- unique(exclusie)

remove(geneesmiddelen)

### LOAD ALL geneesmiddelen

for(f in list.files("../data/geneesmiddelen", pattern=".xlsx", full.names=TRUE)) {

  if (exists("geneesmiddelen")){

    geneesmiddelen <<-rbind(geneesmiddelen,read_xlsx(

      path = f, na = "NULL", skip = 0, col_names = 'words'))

  } else {

    geneesmiddelen <<-read_xlsx(

      path = f, na = "NULL", skip = 0, col_names = 'words')

  }

}

for (e in 1:nrow(geneesmiddelen)){

  geneesmiddelen$words[e] <-gsub("(", "",geneesmiddelen$words[e], fixed=TRUE)

  geneesmiddelen$words[e] <-gsub(")", "",geneesmiddelen$words[e], fixed=TRUE)

  geneesmiddelen$words[e] <-gsub("\\", ";",geneesmiddelen$words[e], fixed=TRUE)

  geneesmiddelen$words[e] <- tolower(geneesmiddelen$words[e])

}

geneesmiddelen <- unique(geneesmiddelen)

remove(intolerantie)

### LOAD ALL intolerantie

for(f in list.files("../data/intolerantie", pattern=".xlsx", full.names=TRUE)) {

  if (exists("intolerantie")){

    intolerantie <<-rbind(intolerantie,read_xlsx(

      path = f, na = "NULL", skip = 0, col_names = 'words'))

  } else {

    intolerantie <<-read_xlsx(

      path = f, na = "NULL", skip = 0, col_names = 'words')

  }

}

remove(medicamenteus)

### LOAD ALL medicamenteus

for(f in list.files("../data/medicamenteus", pattern=".xlsx", full.names=TRUE)) {

  if (exists("medicamenteus")){

    medicamenteus <<-rbind(medicamenteus,read_xlsx(

      path = f, na = "NULL", skip = 0, col_names = 'words'))

  } else {

    medicamenteus <<-read_xlsx(

      path = f, na = "NULL", skip = 0, col_names = 'words')

  }

}

remove(bijwoorden)

### LOAD ALL bijwoorden

for(f in list.files("../data/bijwoorden", pattern=".xlsx", full.names=TRUE)) {

  if (exists("bijwoorden")){

    bijwoorden <<-rbind(bijwoorden,read_xlsx(

      path = f, na = "NULL", skip = 0, col_names = 'words'))

  } else {

    bijwoorden <<-read_xlsx(

      path = f, na = "NULL", skip = 0, col_names = 'words')

  }

}

remove(bijwerking)

### LOAD ALL bijwerking

for(f in list.files("../data/bijwerking", pattern=".xlsx", full.names=TRUE)) {

  if (exists("bijwerking")){

    bijwerking <<-rbind(bijwerking,read_xlsx(

      path = f, na = "NULL", skip = 0, col_names = 'words'))

  } else {

    bijwerking <<-read_xlsx(

      path = f, na = "NULL", skip = 0, col_names = 'words')

  }

}

############# DATA PREP AGAIN ##########################################

data <- df_combined[c("PatientNr", "ADRid", "mergedText", "label")]

data$findFlag <- ""

data$keyword <- ""

names(data) <- c("PatientNr", "ADRid", "opmerking", "label", "findFlag", "keyword")

f = function(x) {

  i <<- i+1

  print(i)

  print("--------------------------------------------------------")

  x <- data.frame(t(x))

  x <- data.frame(lapply(x, as.character), stringsAsFactors=FALSE)

  s <-str_replace_all(x['opmerking'], "\n", " ")

  # print(s)

  s <-str_replace_all(s, "[.]", ";")

  s <-str_replace_all(s, "[,]", ";")

  s <-str_replace_all(s, "[:]", ";")

  s <-str_replace_all(s, "[?]", ";")

  s <-str_replace_all(s, "[(]", ";")

  s <-str_replace_all(s, "[)]", ";")

  s <-gsub("\\", ";",s, fixed=TRUE)

  s <-str_replace_all(s, "(;[ ]+)", "; ")

  s <-str_replace_all(s, "#;", "#")

  s <- tolower(s)

  x['opmerking'] <- s

  r <- data.frame()

  ###VOORZETSELS MED COMBI

  startsList <- array(numeric(),c(0))

  endsList <- array(numeric(),c(0))

  for (j in 1:nrow(geneesmiddelen)) {

    starts <- -1

    searchWord <- geneesmiddelen$words[j]

    if(nchar(searchWord) < 4) {

      searchWord <- paste0(" ",geneesmiddelen$words[j]," ",sep="",collapse=NULL)

    }

    loc <- gregexpr(searchWord, s)[[1]]

    starts <- `attributes<-`(loc,NULL)

    ends <- `attributes<-`(loc,NULL)

    #

    if(starts >= 0){

      for(l in 1:length(starts)){

        ends[l] <- starts[l] + nchar(searchWord) - 1

      }

      startsList <- c(startsList,starts)

      endsList <- c(endsList,ends)

    }

    if(nchar(geneesmiddelen$words[j]) < 4) {

      searchWord <- paste0(" ",geneesmiddelen$words[j],";",sep="",collapse=NULL)

      loc <- gregexpr(searchWord, s)[[1]]

      starts <- `attributes<-`(loc,NULL)

      ends <- `attributes<-`(loc,NULL)

      if(starts >= 0){

        for(l in 1:length(starts)){

          ends[l] <- starts[l] + nchar(searchWord) - 1

        }

        startsList <- c(startsList,starts)

        endsList <- c(endsList,ends)

      }

    }

  }

  medstartList <- startsList

  medendList <- endsList

  startsList <- array(numeric(),c(0))

  endsList <- array(numeric(),c(0))

  lengthWordList <- array(numeric(),c(0))

  for (j in 1:nrow(bijwoorden)) {

    searchWord <- paste(bijwoorden$words[j]," ",sep="",collapse=NULL)

    loc <- gregexpr(searchWord, s)[[1]]

    starts <- `attributes<-`(loc,NULL)

    ends <- `attributes<-`(loc,NULL)

    if(starts == 1){

      for(l in 1:length(starts)){

        starts[l] <- starts[l]

        ends[l] <- starts[l] + nchar(searchWord) - 2

      }

      startsList <- c(startsList,starts)

      endsList <- c(endsList,ends)

    }

    searchWord <- paste(" ",bijwoorden$words[j]," ",sep="",collapse=NULL)

    loc <- gregexpr(searchWord, s)[[1]]

    starts <- `attributes<-`(loc,NULL)

    ends <- `attributes<-`(loc,NULL)

    if(starts >= 0){

      # print(searchWord)

      for(l in 1:length(starts)){

        starts[l] <- starts[l] + 1

        ends[l] <- starts[l] + nchar(searchWord) - 3

      }

      startsList <- c(startsList,starts)

      endsList <- c(endsList,ends)

    }

    searchWord <- paste(";",bijwoorden$words[j]," ",sep="",collapse=NULL)

    loc <- gregexpr(searchWord, s)[[1]]

    starts <- `attributes<-`(loc,NULL)

    ends <- `attributes<-`(loc,NULL)

    if(starts >= 0){

      for(l in 1:length(starts)){

        starts[l] <- starts[l] + 1

        ends[l] <- starts[l] + nchar(searchWord) - 3

      }

      startsList <- c(startsList,starts)

      endsList <- c(endsList,ends)

    }

    searchWord <- paste(" ",bijwoorden$words[j],";",sep="",collapse=NULL)

    loc <- gregexpr(searchWord, s)[[1]]

    starts <- `attributes<-`(loc,NULL)

    ends <- `attributes<-`(loc,NULL)

    if(starts >= 0){

      for(l in 1:length(starts)){

        starts[l] <- starts[l] + 1

        ends[l] <- starts[l] + nchar(searchWord) - 3

      }

      startsList <- c(startsList,starts)

      endsList <- c(endsList,ends)

    }

    searchWord <- paste(";",bijwoorden$words[j],";",sep="",collapse=NULL)

    loc <- gregexpr(searchWord, s)[[1]]

    starts <- `attributes<-`(loc,NULL)

    ends <- `attributes<-`(loc,NULL)

    if(starts >= 0){

      for(l in 1:length(starts)){

        starts[l] <- starts[l] + 1

        ends[l] <- starts[l] + nchar(searchWord) - 3

      }

      startsList <- c(startsList,starts)

      endsList <- c(endsList,ends)

    }

  }

  voorzetselstartList <- startsList

  voorzetselendList <- endsList

  if(length(voorzetselstartList)>0 & length(medstartList)>0){

    for( mi in 1:length(medstartList)){

      for ( vi in 1:length(voorzetselstartList)){

        if ((medstartList[mi] - voorzetselendList[vi] < 17 & medstartList[mi] - voorzetselendList[vi] >= 0) | (voorzetselstartList[vi] - medendList[mi] < 17 & voorzetselstartList[vi] - medendList[mi] >= 0)){

          x['findFlag'] <- 'voorzetsel'

          x['keyword'] <- substr(s, voorzetselstartList[vi], voorzetselendList[vi])

          x['keywordMED'] <- substr(s, medstartList[mi], medendList[mi])

          x['labelText'] <- substr(s, (voorzetselstartList[vi]-50), (voorzetselstartList[vi]+50))

          r <- rbind(r,x)

        }

      }

    }

  }

  for (w in 1:nrow(exclusie)) {

    s <-str_replace_all(s, paste0('',exclusie$words[w],''), "")

  }

  voorzetselstartList <- array(numeric(),c(0))

  medstartList <- array(numeric(),c(0))

  Allergie_woorden <- c("all","alerg","a;","a ;") #TODO: a; is not working well on this sample

  df <- as.data.frame(matrix(Allergie_woorden, ncol = 1, byrow = TRUE))

  names(df) <- c("allergie")

  df <- data.frame(lapply(df, as.character), stringsAsFactors=FALSE)

  startsList <- array(numeric(),c(0))

  for (j in 1:nrow(df)) {

    loc <- gregexpr(df$allergie[j], s)[[1]]

    starts <- `attributes<-`(loc,NULL)

    if (starts >= 0) {

      x['findFlag'] <- 'allergie'

      startsList <- c(startsList,starts)

      for (i in starts){

        x['keyword'] <- df$allergie[j]

        x['keywordMED'] <- ""

        x['labelText'] <- substr(s, (i-50), (i+50))

        r <- rbind(r,x)

      }

    }

  }

  allergiestartList <- startsList

  df <- medicamenteus

  names(df) <- c("medicamenteus")

  s <-str_replace_all(s, ";", " ")

  # print(s)

  startsList <- array(numeric(),c(0))

  for (j in 1:nrow(df)) {

    loc <- gregexpr(df$medicamenteus[j], s)[[1]]

    starts <- `attributes<-`(loc,NULL)

    if(starts >= 0){

      startsList <- c(startsList,starts)

      x['findFlag'] <- 'Medicamenteus'

      for (i in starts){

        x['keyword'] <- df$medicamenteus[j]

        x['keywordMED'] <- ""

        x['labelText'] <- substr(s, (i-50), (i+50))

        r <- rbind(r,x)

      }

    }

  }

  medicstartList <- startsList

  df <- bijwerking

  names(df) <- c("bijwerking")

  startsList <- array(numeric(),c(0))

  for (j in 1:nrow(df)) {

    loc <- gregexpr(df$bijwerking[j], s)[[1]]

    starts <- `attributes<-`(loc,NULL)

    if(starts >= 0){

      startsList <- c(startsList,starts)

      x['findFlag'] <- 'bijwerking'

      for (i in starts){

        x['keyword'] <- df$bijwerking[j]

        x['keywordMED'] <- ""

        x['labelText'] <- substr(s, (i-50), (i+50))

        r <- rbind(r,x)

      }

    }

  }

  bijwerkingstartList <- startsList

  r

}

i <<- 0

data2 <- apply(data, 1, f)

resGS <- data.frame()

for(d in data2){

  resGS <- rbind(resGS,d)

}

resNew <- res

write.csv(res, "result_final.csv", row.names = FALSE)

# unique patient, ADRid that we found

dataCount <- unique(data[c('PatientNr', 'ADRid', "label")])

dataCount <- dataCount[c('label')]

dataCount$count <- 1

dataCount <- aggregate(count ~ label, data = dataCount, FUN = sum, na.rm = TRUE)

unique_found <- unique(resGS[c('PatientNr', 'ADRid')])

unique_found$count <- 1

unique_found <- merge(data, unique_found, by=c('PatientNr', 'ADRid'), all.x = TRUE)

unique_found[is.na(unique_found$count),]$count <- 0

not_found <- unique_found[unique_found$count == 0,]

not_found <- not_found[c("PatientNr", "ADRid", "opmerking", "label")]

unique_not_found <- unique(not_found)

not_found_TP_GASTON <- unique_not_found[unique_not_found$label=='TP_GASTON',]

View(unique_not_found[unique_not_found$label=='TP_GASTON',])

write.csv(not_found, "not_found_final.csv", row.names = FALSE)

# NOT found by us

not_found <- unique(not_found[c('PatientNr', 'ADRid', 'label')])

not_found$count <- 1

not_counts <- aggregate(count ~ label, data = not_found, FUN = sum, na.rm = TRUE)

# Unique patient adrID found

unique_found <- unique(resGS[c('PatientNr', 'ADRid', 'label')])

unique_found$count <- 1

counts <- aggregate(count ~ label, data = unique_found, FUN = sum, na.rm = TRUE)

dataCount <- merge(counts, dataCount, by='label', all.x = TRUE)

dataCount <- merge(dataCount, not_counts, by='label', all.x = TRUE)

colnames(dataCount) <- c('label', 'US', 'GASTON+GOLDEN', 'MISSING')

View(dataCount)

res$count <- 1

counts <- aggregate(count ~ label, data = res, FUN = sum, na.rm = TRUE)

counts$PatientNr <- as.numeric(counts$PatientNr)

counts$ADRid <- as.numeric(counts$ADRid)

counts <- counts[order(counts$ADRid),]

counts <- counts[order(counts$PatientNr),]

row.names(counts) <- NULL

write.csv(counts, "counts.csv", row.names = FALSE)

counts

true <- data[c('PatientNr','ADRid','label')]

true$PatientNr <- as.numeric(true$PatientNr)

true$ADRid <- as.numeric(true$ADRid)

mergedTrue <- merge(true, counts, all.x=TRUE)

mergedTrue$count[mergedTrue$count>0] <- TRUE

mergedTrue$count[is.na(mergedTrue$count)] <- FALSE

mergedTrue$label[mergedTrue$label == 'TP_REVIEW'] <- 1

mergedTrue$label[mergedTrue$label == 'FP_gaston'] <- 0

mergedTrue$label <- as.numeric(mergedTrue$label)

xtabs(~ count + label, data = mergedTrue)

i <<- 0

dataG <- apply(not_found_TP_GASTON, 1, f)

resG <- data.frame()

for(d in dataG){

  resG <- rbind(resG,d)

}

**Final R-algorithm:**

library(pillar)

library(pacman)

library(tidyr)

library(dplyr)

library(ggplot2)

library(ggfortify)

library(caret)

library(lubridate)

library(readxl)

library(tm)

library(stringr)

library(plyr)

library(tibble)

library(stringdist)

library(stringr)

library(RColorBrewer)

library(zoo)

library(readxl)

library(stringdist)

f <- "../data/Database_new.xlsx"

database <<-read_xlsx( path = f, na = "NULL", skip = 0, sheet="Database R")

database <- database[0:292,]

database['ID'] <- seq.int(nrow(database))

data <- database

### LOAD severe ADR list

severe <<-read_xlsx(

  path = "../data/meddra-severe.xlsx", na = "NULL", skip = 0)

### LOAD ALL geneesmiddelen

for(f in list.files("../data/geneesmiddelen", pattern=".xlsx", full.names=TRUE)) {

  if (exists("geneesmiddelen")){

    geneesmiddelen <<-rbind(geneesmiddelen,read_xlsx(

      path = f, na = "NULL", skip = 0, col_names = 'words'))

  } else {

    geneesmiddelen <<-read_xlsx(

      path = f, na = "NULL", skip = 0, col_names = 'words')

  }

}

for (e in 1:nrow(geneesmiddelen)){

  geneesmiddelen$words[e] <-gsub("(", "",geneesmiddelen$words[e], fixed=TRUE)

  geneesmiddelen$words[e] <-gsub(")", "",geneesmiddelen$words[e], fixed=TRUE)

  geneesmiddelen$words[e] <-gsub("\\", "\\\\",geneesmiddelen$words[e], fixed=TRUE)

  geneesmiddelen$words[e] <- tolower(geneesmiddelen$words[e])

}

geneesmiddelen <- unique(geneesmiddelen)

llt <- read_xlsx(path = "../data/LLT.xlsx", na = "NULL", skip = 0)

llt <- llt[c('llt_code', 'llt_name', 'pt_code')]

for (e in 1:nrow(llt)){

  llt$llt_name[e] <- tolower(llt$llt_name[e])

}

mdhier <- read_xlsx(path = "../data/MDHIER.xlsx", na = "NULL", skip = 0)

mdhier <- mdhier[c("pt_code","hlt_code","hlgt_code","soc_code","pt_name","hlt_name","hlgt_name","soc_name","soc_abbrev")]

# Remove SOC codes that are not included.

# Aangelegenheden met betrekking tot producten

# Sociale omstandigheden

# Chirurgische en medische verrichtingen

unique(mdhier$soc_name)

mdhier <- mdhier[!(mdhier$soc_name %in% c('Aangelegenheden met betrekking tot producten', 'Sociale omstandigheden', 'Chirurgische en medische verrichtingen', 'Onderzoeken')),]

unique(mdhier$soc_name)

lltpt <- merge(llt, mdhier, by= "pt_code", all = TRUE)

duplicateNames <- llt[duplicated(llt$llt_name),]

data$ID <- rownames(data)

data$findFlag <- NULL

data$keyword <- NULL

data$keywordMED <- ""

data$startIndexMED <- ""

data$lengthMED <- ""

findGeneesmiddel = function(x) {

  i <<- i+1

  # uncomment if you want to see progress printed

  # print(i)

  # print("--------------------------------------------------------")

  x <- data.frame(t(x))

  x <- data.frame(lapply(x, as.character), stringsAsFactors=FALSE)

  s <-str_replace_all(x['opmerking'], "\n", " ")

  s <-str_replace_all(s, "[.]", ";")

  s <-str_replace_all(s, "[,]", ";")

  s <-str_replace_all(s, "[:]", ";")

  s <-str_replace_all(s, "[?]", ";")

  s <-gsub("\\", ";",s, fixed=TRUE)

  s <-str_replace_all(s, "(;[ ]+)", "; ")

  s <- tolower(s)

  x['opmerking'] <- s

  r <- data.frame()

  ###VOORZETSELS MED COMBI

  startsList <- array(numeric(),c(0))

  for (j in 1:nrow(geneesmiddelen)) {

    starts <- -1

    searchWord <- paste0(" ",geneesmiddelen$words[j]," ")

    loc <- gregexpr(searchWord, s)[[1]]

    starts <- `attributes<-`(loc,NULL)

    if(starts >= 0){

      for( m in starts){

        x['startIndexMED'] <- m

        n_search <- nchar(searchWord)

        x['lengthMED'] <- n_search

        x['keywordMED'] <- searchWord

        r <- rbind(r,x)

      }

    }

    starts <- -1

    searchWord <- paste0(";",geneesmiddelen$words[j]," ")

    loc <- gregexpr(searchWord, s)[[1]]

    starts <- `attributes<-`(loc,NULL)

    if(starts >= 0){

      for( m in starts){

        x['startIndexMED'] <- m

        n_search <- nchar(searchWord)

        x['lengthMED'] <- n_search

        x['keywordMED'] <- searchWord

        r <- rbind(r,x)

      }

    }

    starts <- -1

    searchWord <- paste0(" ",geneesmiddelen$words[j],";")

    loc <- gregexpr(searchWord, s)[[1]]

    starts <- `attributes<-`(loc,NULL)

    if(starts >= 0){

      for( m in starts){

        x['startIndexMED'] <- m

        n_search <- nchar(searchWord)

        x['lengthMED'] <- n_search

        x['keywordMED'] <- searchWord

        r <- rbind(r,x)

      }

    }

    starts <- -1

    searchWord <- paste0(";",geneesmiddelen$words[j],";")

    loc <- gregexpr(searchWord, s)[[1]]

    starts <- `attributes<-`(loc,NULL)

    if(starts >= 0){

      for( m in starts){

        x['startIndexMED'] <- m

        n_search <- nchar(searchWord)

        x['lengthMED'] <- n_search

        x['keywordMED'] <- searchWord

        r <- rbind(r,x)

      }

    }

    starts <- -1

    searchWord <- paste0("",geneesmiddelen$words[j]," ")

    loc <- gregexpr(searchWord, s)[[1]]

    starts <- `attributes<-`(loc,NULL)

    if(starts == 1){

      for( m in starts){

        x['startIndexMED'] <- m

        n_search <- nchar(searchWord)

        x['lengthMED'] <- n_search

        x['keywordMED'] <- searchWord

        r <- rbind(r,x)

      }

    }

  }

  r

}

#Function to find MEDDRA TERMS

findMeddra = function(x) {

  i <<- i+1

  print(i)

  print("--------------------------------------------------------")

  x <- data.frame(t(x))

  x <- data.frame(lapply(x, as.character), stringsAsFactors=FALSE)

  s <-str_replace_all(x['opmerking'], "\n", " ")

  s <-str_replace_all(s, "[.]", ";")

  s <-str_replace_all(s, "[,]", ";")

  s <-str_replace_all(s, "[:]", ";")

  s <-str_replace_all(s, "[?]", ";")

  s <-str_replace_all(s, "[(]", ";")

  s <-str_replace_all(s, "[)]", ";")

  s <-str_replace_all(s, "[-]", ";")

  s <-gsub("\\", ";",s, fixed=TRUE)

  s <-str_replace_all(s, "(;[ ]+)", "; ")

  s <- tolower(s)

  x['opmerking'] <- s

  r <- data.frame()

  for (j in 1:nrow(llt)) {

    starts <- -1

    searchWord <- paste0(" ",llt$llt_name[j]," ")

    loc <- gregexpr(searchWord, s)[[1]]

    starts <- `attributes<-`(loc,NULL)

    if(starts >= 0 & nchar(searchWord)>3){

      for( m in starts){

        x['startIndex_llt_name'] <- m

        x['length_llt_name'] <- nchar(searchWord)

        x['llt_name'] <- llt$llt_name[j]

        x['llt_code'] <- llt$llt_code[j]

        x['pt_code'] <- llt$pt_code[j]

        x['pt_name'] <- ""

        r <- rbind(r,x)

      }

    }

  }

  for (j in 1:nrow(llt)) {

    starts <- -1

    searchWord <- paste0(" ",llt$llt_name[j],";")

    loc <- gregexpr(searchWord, s)[[1]]

    starts <- `attributes<-`(loc,NULL)

    if(starts >= 0 & nchar(searchWord)>3){

      for( m in starts){

        x['startIndex_llt_name'] <- m

        x['length_llt_name'] <- nchar(searchWord)

        x['llt_name'] <- llt$llt_name[j]

        x['llt_code'] <- llt$llt_code[j]

        x['pt_code'] <- llt$pt_code[j]

        x['pt_name'] <- ""

        r <- rbind(r,x)

      }

    }

  }

  for (j in 1:nrow(llt)) {

    starts <- -1

    searchWord <- paste0(";",llt$llt_name[j]," ")

    loc <- gregexpr(searchWord, s)[[1]]

    starts <- `attributes<-`(loc,NULL)

    if(starts >= 0 & nchar(searchWord)>3){

      for( m in starts){

        x['startIndex_llt_name'] <- m

        x['length_llt_name'] <- nchar(searchWord)

        x['llt_name'] <- llt$llt_name[j]

        x['llt_code'] <- llt$llt_code[j]

        x['pt_code'] <- llt$pt_code[j]

        x['pt_name'] <- ""

        r <- rbind(r,x)

      }

    }

  }

  for (j in 1:nrow(llt)) {

    starts <- -1

    searchWord <- paste0(";",llt$llt_name[j],";")

    loc <- gregexpr(searchWord, s)[[1]]

    starts <- `attributes<-`(loc,NULL)

    if(starts >= 0 & nchar(searchWord)>3){

      for( m in starts){

        x['startIndex_llt_name'] <- m

        x['length_llt_name'] <- nchar(searchWord)

        x['llt_name'] <- llt$llt_name[j]

        x['llt_code'] <- llt$llt_code[j]

        x['pt_code'] <- llt$pt_code[j]

        x['pt_name'] <- ""

        r <- rbind(r,x)

      }

    }

  }

  for (j in 1:nrow(llt)) {

    starts <- -1

    searchWord <- paste0("",llt$llt_name[j]," ")

    loc <- gregexpr(searchWord, s)[[1]]

    starts <- `attributes<-`(loc,NULL)

    if(starts == 1 & nchar(searchWord)>3){

      for( m in starts){

        x['startIndex_llt_name'] <- m

        x['length_llt_name'] <- nchar(searchWord)

        x['llt_name'] <- llt$llt_name[j]

        x['llt_code'] <- llt$llt_code[j]

        x['pt_code'] <- llt$pt_code[j]

        x['pt_name'] <- ""

        r <- rbind(r,x)

      }

    }

  }

  for (j in 1:nrow(mdhier)) {

    #SPACES

    starts <- -1

    searchWord <- paste0(" ",mdhier$pt_name[j]," ")

    loc <- gregexpr(searchWord, s)[[1]]

    starts <- `attributes<-`(loc,NULL)

    if(starts >= 0){

      for( m in starts){

        x['startIndex_llt_name'] <- m

        x['length_llt_name'] <- nchar(searchWord)

        x['llt_name'] <- ""

        x['llt_code'] <- ""

        x['pt_code'] <- mdhier$pt_code[j]

        x['pt_name'] <- mdhier$pt_name[j]

        r <- rbind(r,x)

      }

    }

    #BEGIN TEXT

    starts <- -1

    searchWord <- paste(mdhier$pt_name[j]," ",sep="",collapse=NULL)

    loc <- gregexpr(searchWord, s)[[1]]

    starts <- `attributes<-`(loc,NULL)

    if(starts == 1){

      for( m in starts){

        x['startIndex_llt_name'] <- m

        x['length_llt_name'] <- nchar(searchWord)

        x['llt_name'] <- ""

        x['llt_code'] <- ""

        x['pt_code'] <- mdhier$pt_code[j]

        x['pt_name'] <- mdhier$pt_name[j]

        r <- rbind(r,x)

      }

    }

    starts <- -1

    searchWord <- paste0(";",mdhier$pt_name[j]," ")

    loc <- gregexpr(searchWord, s)[[1]]

    starts <- `attributes<-`(loc,NULL)

    if(starts >= 0){

      for( m in starts){

        x['startIndex_llt_name'] <- m

        x['length_llt_name'] <- nchar(searchWord)

        x['llt_name'] <- ""

        x['llt_code'] <- ""

        x['pt_code'] <- mdhier$pt_code[j]

        x['pt_name'] <- mdhier$pt_name[j]

        r <- rbind(r,x)

      }

    }

    starts <- -1

    searchWord <- paste0(" ",mdhier$pt_name[j],";")

    loc <- gregexpr(searchWord, s)[[1]]

    starts <- `attributes<-`(loc,NULL)

    if(starts >= 0){

      for( m in starts){

        x['startIndex_llt_name'] <- m

        x['length_llt_name'] <- nchar(searchWord)

        x['llt_name'] <- ""

        x['llt_code'] <- ""

        x['pt_code'] <- mdhier$pt_code[j]

        x['pt_name'] <- mdhier$pt_name[j]

        r <- rbind(r,x)

      }

    }

    starts <- -1

    searchWord <- paste0(";",mdhier$pt_name[j],";")

    loc <- gregexpr(searchWord, s)[[1]]

    starts <- `attributes<-`(loc,NULL)

    if(starts >= 0){

      for( m in starts){

        x['startIndex_llt_name'] <- m

        x['length_llt_name'] <- nchar(searchWord)

        x['llt_name'] <- ""

        x['llt_code'] <- ""

        x['pt_code'] <- mdhier$pt_code[j]

        x['pt_name'] <- mdhier$pt_name[j]

        r <- rbind(r,x)

      }

    }

  }

  r

}

findGeneesmiddelLevenshtein = function(x) {

  i <<- i+1

  print(i)

  print("--------------------------------------------------------")

  x <- data.frame(t(x))

  x <- data.frame(lapply(x, as.character), stringsAsFactors=FALSE)

  s <-str_replace_all(x['opmerking'], "\n", " ")

  s <-str_replace_all(s, "[.]", ";")

  s <-str_replace_all(s, "[,]", ";")

  s <-str_replace_all(s, "[:]", ";")

  s <-str_replace_all(s, "[?]", ";")

  s <-str_replace_all(s, "[/]", ";")

  s <-str_replace_all(s, "[(]", ";")

  s <-str_replace_all(s, "[)]", ";")

  s <-gsub("\\", ";",s, fixed=TRUE)

  s <-str_replace_all(s, "(;[ ]+)", "; ")

  s <- tolower(s)

  x['opmerking'] <- s

  r <- data.frame()

  ###VOORZETSELS MED COMBI

  startsList <- array(numeric(),c(0))

  s <-str_replace_all(s, "[;]", " ")

  s <-str_replace_all(s, "([ ]+)", " ")

  sl <- strsplit(s, c(" ",";"),TRUE)[[1]]

  foundList <- c()

  for (word in sl){

    for (j in 1:nrow(geneesmiddelen)) {

      if (nchar(word) > 4 && stringdist(word,geneesmiddelen$words[j],"lv") < 2){

        foundList <- unlist(c(foundList, word))

      }

    }

  }

  for(searchword in foundList){

    starts <- -1

    searchWord <- paste0(" ",searchword," ")

    #searchWord <- geneesmiddelen$words[j]

    loc <- gregexpr(searchWord, s)[[1]]

    starts <- `attributes<-`(loc,NULL)

    if(starts >= 0){

      for( m in starts){

        x['startIndexMED'] <- m

        n_search <- nchar(searchWord)

        x['lengthMED'] <- n_search

        x['keywordMED'] <- searchWord

        r <- rbind(r,x)

      }

    }

  }

  r

}

findNegationsLevenshtein = function(x) {

  i <<- i+1

  print(i)

  print("--------------------------------------------------------")

  x <- data.frame(t(x))

  x <- data.frame(lapply(x, as.character), stringsAsFactors=FALSE)

  s <-str_replace_all(x['opmerking'], "\n", " ")

  s <-str_replace_all(s, "[.]", ";")

  s <-str_replace_all(s, "[,]", ";")

  s <-str_replace_all(s, "[:]", ";")

  s <-str_replace_all(s, "[?]", ";")

  s <-str_replace_all(s, "[/]", ";")

  s <-str_replace_all(s, "[(]", ";")

  s <-str_replace_all(s, "[)]", ";")

  s <-gsub("\\", ";",s, fixed=TRUE)

  s <-str_replace_all(s, "(;[ ]+)", "; ")

  s <- tolower(s)

  startsList <- array(numeric(),c(0))

  s <-str_replace_all(s, "[;]", " ")

  for ( searchword in negationList ) {

    starts <- -1

    searchWord <- paste0(" ",searchword," ")

    loc <- gregexpr(searchWord, s)[[1]]

    starts <- `attributes<-`(loc,NULL)

    if ( starts >= 0 ) {

      for ( m in starts ) {

        x['startIndexNeg'] <- m

        n_search <- nchar(searchWord)

        x['lengthNeg'] <- n_search

        x['keywordNeg'] <- searchWord

        r <- rbind(r,x)

      }

    }

  }

  r

}

findNegations = function(x) {

  i <<- i+1

  print(i)

  print("--------------------------------------------------------")

  x <- data.frame(t(x))

  x <- data.frame(lapply(x, as.character), stringsAsFactors=FALSE)

  s <-str_replace_all(x['opmerking'], "\n", " ")

  s <-str_replace_all(s, "[.]", ";")

  s <-str_replace_all(s, "[,]", ";")

  s <-str_replace_all(s, "[:]", ";")

  s <-str_replace_all(s, "[?]", ";")

  s <-str_replace_all(s, "[/]", ";")

  s <-str_replace_all(s, "[(]", ";")

  s <-str_replace_all(s, "[)]", ";")

  s <-gsub("\\", ";",s, fixed=TRUE)

  s <-str_replace_all(s, "(;[ ]+)", "; ")

  s <- tolower(s)

  startsList <- array(numeric(),c(0))

  s <-str_replace_all(s, "[;]", " ")

  for ( searchword in negationList ) {

    starts <- -1

    searchWord <- paste0(" ",searchword," ")

    loc <- gregexpr(searchWord, s)[[1]]

    starts <- `attributes<-`(loc,NULL)

    if ( starts >= 0 ) {

      for ( m in starts ) {

        x['startIndexNeg'] <- m

        n_search <- nchar(searchWord)

        x['lengthNeg'] <- n_search

        x['keywordNeg'] <- searchWord

        r <- rbind(r,x)

      }

    }

  }

  r

}

# Function to find bijworden

# Only used for phase 1

findBijwoord = function(x) {

  i <<- i+1

  print(i)

  print("--------------------------------------------------------")

  x <- data.frame(t(x))

  x <- data.frame(lapply(x, as.character), stringsAsFactors=FALSE)

  s <-str_replace_all(x['opmerking'], "\n", " ")

  s <-str_replace_all(s, "[.]", ";")

  s <-str_replace_all(s, "[,]", ";")

  s <-str_replace_all(s, "[:]", ";")

  s <-str_replace_all(s, "[?]", ";")

  s <-gsub("\\", ";",s, fixed=TRUE)

  s <-str_replace_all(s, "(;[ ]+)", "; ")

  s <- tolower(s)

  x['opmerking'] <- s

  r <- data.frame()

  for (j in 1:nrow(bijwoorden)) {

    searchWord <- paste(bijwoorden$words[j]," ",sep="",collapse=NULL)

    loc <- gregexpr(searchWord, s)[[1]]

    starts <- `attributes<-`(loc,NULL)

    if(starts == 1){

      for(l in 1:length(starts)){

        starts[l] <- starts[l] + nchar(searchWord)-1

      }

      startsList <- c(startsList,starts)

    }

    searchWord <- paste(" ",bijwoorden$words[j],"-",sep="",collapse=NULL)

    loc <- gregexpr(searchWord, s)[[1]]

    starts <- `attributes<-`(loc,NULL)

    if(starts >= 0){

      for(l in 1:length(starts)){

        starts[l] <- starts[l] + nchar(searchWord)-1

      }

      startsList <- c(startsList,starts)

    }

    searchWord <- paste(" ",bijwoorden$words[j]," ",sep="",collapse=NULL)

    loc <- gregexpr(searchWord, s)[[1]]

    starts <- `attributes<-`(loc,NULL)

    if(starts >= 0){

      for(l in 1:length(starts)){

        starts[l] <- starts[l] + nchar(searchWord)-1

      }

      startsList <- c(startsList,starts)

    }

    searchWord <- paste(";",bijwoorden$words[j]," ",sep="",collapse=NULL)

    loc <- gregexpr(searchWord, s)[[1]]

    starts <- `attributes<-`(loc,NULL)

    if(starts >= 0){

      for(l in 1:length(starts)){

        starts[l] <- starts[l] + nchar(searchWord)-1

      }

      startsList <- c(startsList,starts)

    }

    searchWord <- paste(" ",bijwoorden$words[j],";",sep="",collapse=NULL)

    loc <- gregexpr(searchWord, s)[[1]]

    starts <- `attributes<-`(loc,NULL)

    if(starts >= 0){

      for(l in 1:length(starts)){

        starts[l] <- starts[l] + nchar(searchWord)-1

      }

      startsList <- c(startsList,starts)

    }

    searchWord <- paste(";",bijwoorden$words[j],";",sep="",collapse=NULL)

    loc <- gregexpr(searchWord, s)[[1]]

    starts <- `attributes<-`(loc,NULL)

    if(starts >= 0){

      for(l in 1:length(starts)){

        starts[l] <- starts[l] + nchar(searchWord)-1

      }

      startsList <- c(startsList,starts)

    }

  }

  r

}

### create database of found medication

i <<- 0

database <- data

foundGeneesmiddelNew <- apply(database, 1, findGeneesmiddel)

resFoundGeneesmiddelNew <- data.frame()

for(d in foundGeneesmiddelNew){

  resFoundGeneesmiddelNew <- rbind(resFoundGeneesmiddelNew, d)

}

### Uncomment if you want to store the medication terms found

# write.csv2(resFoundGeneesmiddelNew, "resFoundGeneesmiddel.csv", row.names = FALSE)

i <<- 0

### Create database of found meddra terms

foundMeddraNew <- apply(database, 1, findMeddra)

resFoundMeddraNew <- data.frame()

for(d in foundMeddraNew){

  resFoundMeddraNew <- rbind(resFoundMeddraNew, d)

}

### Uncomment if you want to store the Meddra terms found

# write.csv2(resFoundMeddraNew, "resFoundMeddra.csv", row.names = FALSE)

###############################################################

# YOU CAN RUN FROM HERE WITHOUT COST

meddracalc <- resFoundMeddraNew[,c("PatientNr","ID","startIndex_llt_name","length_llt_name","llt_name","llt_code","pt_code", "nummer.llT.PT")]

pt <- mdhier[c('pt_code','pt_name', 'soc_name', 'hlgt_name','hlt_name')]

pt <- unique(pt)

#ontdubbelen van gevonden PT?

meddracalc <- merge(meddracalc, pt, by = "pt_code", all.x = TRUE)

meddracalc <- meddracalc[,c("PatientNr","ID","nummer.llT.PT","startIndex_llt_name","length_llt_name","llt_name","pt_code","pt_name", 'soc_name', 'hlgt_name','hlt_name')]

medcalc <- resFoundGeneesmiddelNew

medcalc <- medcalc[,c("ID","startIndexMED","lengthMED","keywordMED")]

#Merge PT and med

res <- merge(meddracalc, medcalc, by="ID")

res <- unique(res)

write.csv2(res, "resFoundGeneesmiddelEnMeddra.csv", row.names = FALSE)

# RUN FROM HERE TO TEST WITH DIFFERENT DELTA

# Find the matching PT and LLT codes of meddra

isMatchCode <- function(x) {

  return(grepl(x['pt_code'], x['nummer.llT.PT'] ))

}

# Return if a ADR is Severe

isSevereTrue <- function(x) {

  isSevere <- FALSE

  split <- strsplit(x['nummer.llT.PT'], " ", fixed=T)

  for( s1 in split ) {

    severeFound <- s1 == severe$Code

    for ( s2 in severeFound ) {

      if( s2 == TRUE ){

        return(TRUE)

      }

    }

  }

  return(FALSE)

}

isSevereFound <- function(x) {

  isSevere <- FALSE

  severeFound <- x['pt_code'] == severe$Code

  for (s in severeFound) {

    if(s == TRUE) {

      return(TRUE)

    }

  }

  return(FALSE)

}

res <- res[!(res$soc_name %in% c('Aangelegenheden met betrekking tot producten', 'Sociale omstandigheden', 'Chirurgische en medische verrichtingen', 'Onderzoeken')),]

res <- res[!(res$hlgt_name %in% c('therapeutische en niet-therapeutische effecten (excl. toxiciteit)', 'voortplantingsstelselaandoeningen NEG', 'letsels NEG', 'diverse en niet plaatsgespecif. neoplasmata, maligne en niet-gespecif.')),]

res <- res[!(res$hlt_name %in% c("hernia's NEG", "paresthesieën en dysesthesieën", "huid en subcutaan weefsel virusinfecties","tand- en peridontiuminfecties en -ontstekingen",

                                 "cerebrovasculaire en ruggenmergnecrose en vasculaire insufficiëntie",

                                 "oesofagitis (excl. infectieus)",

                                 "longen vasculaire aandoeningen NEG",

                                 "longoedemen",

                                 "tussenwervelschijfaandoeningen NEG",

                                 "verhoogde lichamelijke activiteitsniveaus",

                                 "neoplasmata plaats niet-gespecificeerd maligne NEG",

                                 "neoplasmata niet-gespecificeerde maligniteit en plaats niet-gespecificeerd NEG",

                                 "baarmoederhalsaandoeningen NEG",

                                 "Parapox-virusinfecties",

                                 "necrose en vasculaire insufficiëntie plaats niet-gespecificeerd NEG")),]

purine <- res[(res$hlt_name %in% c("afwijkingen van purinemetabolisme")),]

purine <- purine[(purine$pt_code == 10018627),]

res <- res[!(res$hlt_name %in% c("afwijkingen van purinemetabolisme")),] #PT Jicht mag er niet uit moeten we filteren

res <- rbind(res,purine)

res <- res[!(res$pt_name == "pijn"),]

res <- res[!(res$pt_name == "carditis"),]

res <- res[!(res$pt_name == "astma cardiale"),]

res <- res[!(res$llt_name == "diabetisch"),]

res <- res[!(res$llt_name == "ALS"),]

res <- res[!(res$llt_name == "af"),]

res <- res[!(res$pt_name == "af"),]

res <- res[!(res$pt_name == "vd"),]

res <- res[!(res$llt_name == "AF"),]

res <- res[!(res$llt_name == "MDS"),]

res <- res[!(res$pt_code == 10002026),]

res <- res[!(res$pt_code == 10035226),]

res <- res[!(res$pt_code == 10076311),]

res <- res[!(res$pt_code == 10011078),]

res <- res[!(res$pt_code == 10025409),]

res <- res[!(res$pt_code == 10014778),]

res <- res[!(res$pt_code == 10025476),]

res <- res[!(res$pt_code == 10035664),]

res <- res[!(res$pt_code == 10035669),]

res <- res[!(res$llt_name == "roos"),]

res <- res[!(res$llt_name == "sputum"),]

res <- res[!(res$llt_name == "bleek"),]

res <- res[!(res$llt_name == "coagulopathie"),]

res <- res[!(res$llt_name == "mds"),]

res <- res[!(res$llt_name == "ten"),]

res <- res[!(res$llt_name == "coloncarcinoom"),]

res <- res[!(res$llt_name == "copd"),]

res <- res[!(res$llt_name == "cva"),]

res <- res[!(res$llt_name == "vd"),]

res <- res[!(res$llt_name == "pancreascarcinoom"),]

res <- res[!(res$llt_name == "ziekte"),]

res <- res[!(res$llt_name == "em"),]

res <- res[!(res$llt_name == "pac"),]

res <- res[!(res$llt_name == "angst"),]

res <- res[!(res$llt_name == "pericardeffusie"),]

res <- res[!(res$llt_name == "narcose"),]

res <- res[!(res$llt_name == "coloncarcinoom"),]

res <- res[!(res$llt_name == "inactiviteit"),]

res <- res[!(res$llt_name == "lymfoom"),]

res <- res[!(res$llt_name == "ms"),]

res <- res[!(res$llt_name == "tia"),]

res <- drop_na(res)

res$severeTrue <- apply(res, 1 , isSevereTrue)

res$severeFound <- apply(res, 1 , isSevereFound)

resPtFirst <- res[res$startIndex_llt_name < res$startIndexMED,]

resMedFirst <- res[res$startIndex_llt_name > res$startIndexMED,]

resPtFirst$distance <- abs((resPtFirst$startIndex_llt_name+resPtFirst$length_llt_name-1)-resPtFirst$startIndexMED)

resMedFirst$distance <- abs((resMedFirst$startIndexMED+resMedFirst$lengthMED-1)-resMedFirst$startIndex_llt_name)

listSens <- c(0)

listppv <- c(0)

bestDelta <- 0

bestPPV <- 0

sensBestPPV <- 0

for ( delta in 5:100 ) {

  resPtFirst2 <- resPtFirst[ abs((resPtFirst$startIndex_llt_name+resPtFirst$length_llt_name-1)-resPtFirst$startIndexMED)<= delta,]

  resMedFirst2 <- resMedFirst[abs((resMedFirst$startIndexMED+resMedFirst$lengthMED-1)-resMedFirst$startIndex_llt_name) <= delta,]

  resFinal <- rbind(resPtFirst2,resMedFirst2)

  resPatientUnique <- unique(resFinal[!duplicated(resFinal[,c("PatientNr", "keywordMED")]),])

  resPatientUnique$match <- apply(resPatientUnique, 1 , isMatchCode)

  resPatientUnique$count <-  0

  resPatientUnique$countTotal <- 1

  resPatientUnique[resPatientUnique$match==TRUE,]$count <- 1

  ppv <- sum(resPatientUnique$count)/sum(resPatientUnique$countTotal)

  sens <- sum(resPatientUnique$count)/(161)

  listppv <- list(listppv, ppv)

  listSens <- list(listSens, sens)

  if (bestPPV < ppv) {

    bestResPatientUnique <- resPatientUnique

    bestPPV <-  ppv

    sensBestPPV <- sens

    bestDelta <- delta

    resBest <- resFinal

    bestFound <- sum(resPatientUnique$count)

    bestTotal <- sum(resPatientUnique$countTotal)

  }

}

plot(4:100,unlist(listppv))

plot(4:100,unlist(listSens))

write.csv2(bestResPatientUnique, "resPatientUnique.csv", row.names = FALSE)
